# Matrine regulates autophagy in ileal epithelial cells in a porcine circovirus type 2-infected murine model

**DOI:** 10.3389/fmicb.2024.1455049

**Published:** 2024-11-11

**Authors:** Hong Wang, Na Sun, Panpan Sun, Hua Zhang, Wei Yin, Xiaozhong Zheng, Kuohai Fan, Yaogui Sun, Hongquan Li

**Affiliations:** ^1^Shanxi Key Laboratory for Modernization of TCVM, College of Veterinary Medicine, Shanxi Agricultural University, Taigu, Shanxi, China; ^2^Department of Sports, Shanxi Agricultural University, Taigu, Shanxi, China; ^3^Centre for Inflammation Research, Queen’s Medical Research Institute, The University of Edinburgh, Edinburgh, United Kingdom; ^4^Laboratory Animal Center, Shanxi Agricultural University, Taigu, Shanxi, China

**Keywords:** porcine circovirus type 2, matrine, antiviral, mechanical barrier, autophagy

## Abstract

**Introduction:**

Porcine circovirus type 2 (PCV2) is an important pathogen that causes diarrhea in nursery and fattening pigs, resulting in huge economic losses for commercial pig farms. Protective efficacy of vaccines is compromised by mutations in pathogens. There is an urgent need to articulate the mechanism by which PCV2 destroys the host’s intestinal mucosal barrier and to find effective therapeutic drugs. Increasing attention has been paid to the natural antiviral compounds extracted from traditional Chinese medicines. In the present study, we investigated the role of Matrine in mitigating PCV2-induced intestinal damage and enhancing autophagy as a potential therapeutic strategy in mice.

**Methods:**

A total of 40 female, specific-pathogen-free-grade Kunming mice were randomly divided into four groups with 10 mice in each group: control, PCV2 infection, Matrine treatment (40 mg/kg Matrine), and Ribavirin treatment (40 mg/kg Ribavirin). Except for the control group, all mice were injected intraperitoneally with 0.5 mL 10^5.4^ 50% tissue culture infectious dose (TCID_50_)/mL PCV2.

**Results:**

While attenuating PCV2-induced downregulation of ZO-1 and occludin and restoring intestinal barrier function in a PCV2 Kunming mouse model, treatment with Matrine (40 mg/kg) attenuated ultrastructural damage and improved intestinal morphology. Mechanistically, Matrine reversed PCV2-induced autophagosome accumulation by inhibiting signal transducer and activator of transcription 3 (STAT3) phosphorylation and upregulating Beclin1 protein expression, thus resisting viral hijacking of enterocyte autophagy.

**Discussion:**

Our findings demonstrate that Matrine may be a novel, potential antiviral agent against PCV2 by activating intestine cellular autophagy, which provides a new strategy for host-directed drug discovery.

## Graphical abstract

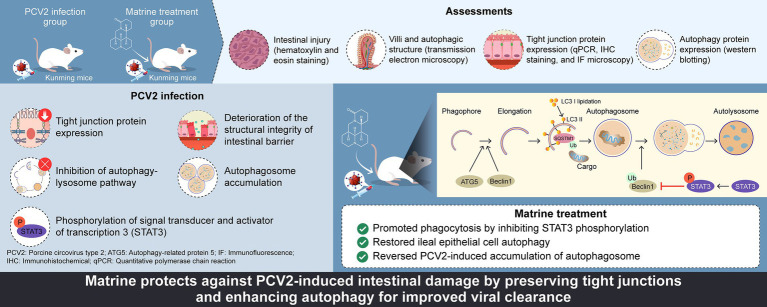

## 1 Introduction

Circovirus (Circoviridae family) is a class of small, envelope-less DNA viruses that are capable of infecting a wide range of vertebrates, including humans and domestic animals. Porcine circovirus type 2 (PCV2) is an important pathogen that causes diarrhea and immunosuppression in pigs at all stages of growth. PCV2 is a generic term for a large group of systemic porcine diseases caused by PCV2 infection that cause substantial economic losses to the global pig industry (Rosell et al., 2000; Wilfred et al., 2018). A positive detection rate of PCV2 in 1,385 samples from pig herds in 15 provinces in China of 57.95% has been reported (Cao et al., 2024). The clinical symptoms of PCV2 enteric disease, one of the main symptoms of PCV2 systemic disease, include diarrhea (mainly watery diarrhea), a decreased feed conversion ratio, growth retardation, weight loss, and increased mortality (Chae, 2005; Segalés, 2012). Therefore, it is important to determine the pathogenic mechanisms of PCV2 intestinal damage in piglets for its prevention and treatment.

The intestinal mucosal barrier is an important line of defense for the body against invading pathogens. Intestinal epithelial cells (IECs) proliferate and renew rapidly to preserve barrier integrity, with tight junction protein complexes representing “gates and fences” that prevent the paracellular transport of bacteria and toxic macromolecules (Marchiando et al., 2010; Spalinger et al., 2021). The imbalance in the homeostasis of IECs and downregulation of tight junction protein complexes compromises the structural integrity of the intestinal mucosal barrier (Chelakkot et al., 2018). The downregulation of the tight junction proteins, claudin and occludin, after a combined infection of porcine bocavirus and PCV2 has been verified in IPEC-J2 cells (Zhang et al., 2018). Zhang et al. (2023) determined the propagation characteristics of PCV2 in ileal and jejunal epithelial cells. No *in vivo* experiments have been performed to study the damaging effects of PCV2 replication on ileal tissue. The correlation between PCV2 infection and the intestinal mucosal barrier is unclear and more in-depth studies are needed to elucidate the relationship.

Autophagy, together with its associated regulatory systems, plays a crucial role in maintaining intestinal integrity and health via tight junction regulation (Foerster et al., 2022). The involvement of autophagy in the initiation and development of PCV2 infections has long been recognized (Gan et al., 2015; Qian et al., 2017; Sinha et al., 2012; Yin et al., 2019; Zhai et al., 2019; Zhu et al., 2012a). Autophagy is an intracellular clearance mechanism that encompasses many highly dynamic biological stages, beginning with autophagosome generation, which is mediated by the combined action of two ubiquitin-like pathways associated with ATG5 and LC3 (Hwang et al., 2012; Yang et al., 2018); and culminating in autophagosome breakdown inside lysosomes, which is marked by the degradation of SQSTM1 (Choi et al., 2013). Beclin1 plays a regulatory role in the recruitment of other autophagy-associated proteins during the initial stages of autophagy and promotes the progression of the autophagy–lysosome pathway by inducing the fusion of autophagosomes and lysosomes (Chen et al., 2020).

Matrine is a quinolizidine alkaloid present in the genus, *Sophora,* of the family, Fabaceae (Li et al., 2020; Li et al., 2021; Zhang et al., 2020). We have previously reported the antiviral activities of Matrine against PCV2, porcine reproductive and respiratory syndrome virus (PRRSV), and encephalomyocarditis virus (Cheng et al., 2013; Xu et al., 2020; Zheng et al., 2021). An amount of 40 mg/kg Matrine inhibited virus replication in the liver and alleviated virus-induced interstitial pneumonia (Sun et al., 2020). Natural products that included Matrine extracted from *Sophora flavescens* Ait. inhibited enterovirus 71 (Jin et al., 2017; Yang et al., 2012), pig epidemic diarrhea virus (Kim et al., 2008), and transmissible gastroenteritis virus (Wang et al., 2017) infection *in vitro*. The expression of tight junction proteins was substantially increased in a Matrine-treated group, which was able to protect the composition and function of the intestinal barrier and was considered to be an effective drug against *Eimeria tenella* (Yuan et al., 2023). In the current study, we found that Matrine inhibited PCV2 infection of the intestine, protected the intestinal barrier function, and promoted intestinal clearance of PCV2 by activating cellular autophagy.

## 2 Materials and methods

### 2.1 Establishment of PCV2 Kunming mouse model and drug treatment

The mice used in this study were procured from Beijing Vital River Laboratory Animal Technology Co., Ltd., Beijing, China. Mice were handled and maintained in accordance with the regulations established by the Institutional Animal Care and Use Committee of Shanxi Agricultural University of Shanxi Agriculture University (ratification number SXAU-EAW-2020 M1130-02). A total of 40 female, specific-pathogen-free (SPF)-grade Kunming mice were randomly allocated to four groups, with 10 animals in each group: control, PCV2 infection (PCV2), Matrine treatment (Matrine), and Ribavirin treatment group (Ribavirin).

Animals in all groups (except the control group) were intraperitoneally injected with 0.5 mL PCV2 toxin (10^5.4^ 50% tissue culture infectious dose, TCID_50_/mL; retained in the laboratory; passaged in PK-15 cells), whereas the mice in the control group received an equivalent amount of saline solution. The intraperitoneal injection of drugs was started on day 5 after injection with PCV2 toxin, and the mice were fasted for 12 h (without water) before medication was administered at a dose of 40 mg/kg body weight for Matrine and Ribavirin (Sun et al., 2020) once daily for 5 d. Mice treated with Ribavirin were used as the positive control group. On the 11th day after PCV2 challenge (1 d after the end of drug administration), the mice were humanely euthanized using cervical dislocation. Ileal tissues of approximately 2 cm in length from the upper part of the ileocecum were collected, snap frozen in liquid nitrogen, and then stored in a − 80°C freezer until further experimental analysis. A portion of the ileal tissue were fixed in Bouin’s fixative (saturated picric acid∶ glacial acetic acid: 40% formaldehyde = 15∶5∶1) for subsequent hematoxylin and eosin (H&E) staining for pathological examination, or fixed in 2.5% glutaraldehyde fixative for transmission electron microscopy (TEM).

### 2.2 Quantification of PCV2 DNA in ileal tissues

Ileal genomic DNA was isolated using a DNA extraction kit (Tiangen, Beijing, China), and the DNA concentration was measured using a NanoDrop 1,000 spectrophotometer (NanoDrop Technologies, Wilmington, DE, United States). Quantitative real-time PCR (qPCR) was used to amplify the DNA fragments using primers for the *Cap* gene of PCV2. The primers listed in Table 1 were used to amplify specific 148-bp fragments of gene transcripts. The qPCR was performed using an Applied Biosystems 7,500 Fast Real-Time PCR System and the 2× SYBR Green qPCR Master Mix (Low ROX, Bimake, United States). A recombinant plasmid vector carrying a portion of the PCV2 *Cap* gene was logarithmically diluted ranging from 10^2^ to 10^8^ copies and was amplified for the construction of a standard curve.

**Table 1:** Primers used for quantitative real-time polymerase chain reaction.

Gene name	Sense primers (5′–3′)	Antisense primers (5′–3′)
*ZO-1*	ACCCGAAACTGATGCTGTGGATAG	AAATGGCCGGGCAGAACTTGTGTA
*Occludin*	TAAGAGCTTACAGGCAGAACTAG	CTGTCATAATCTCCCACCATC
*GADPH*	CCTCGTCCCGTAGACAAAATG	TGAGGTCAATGAAGGGGTCGT

### 2.3 Histomorphological observation of ileal tissue

Small intestinal tissue was preserved, dried using an alcohol gradient series, and embedded in paraffin wax blocks. The sectioning of paraffin-embedded specimens with a thickness of 5 μm was carried out using a microtome (Leica RM2255, Wetzlar, Germany). Sections were deparaffinized using xylene, followed by rehydration from ethanol to water. The sections were then stained with hematoxylin for 7 min. Subsequently, they were rinsed with water and immersed in a series of ethanol solutions of increasing concentration: 70% ethanol for 2 min, 85% ethanol for 2 min, and 90% ethanol for 2 min. The sections were then stained with eosin for 2 min. This was followed by sequential immersions in 95% ethanol for 4 min, 100% ethanol for 5 min, 100% ethanol for 5 min, and xylene for 5 min. Finally, the sections were immersed in xylene for 5 min and sealed using a neutral resin. The mounted slides were analyzed and images were captured using a LEICA DM3000 light emitting diode microscope (Leica Wetzlar, Germany). Sections were observed at ×100 magnification using a bright-field microscope. Measurements of crypt and villus lengths were conducted from 10 representative images for each animal using Image-Pro Plus 6.0 software. The values obtained were averaged for each tissue type. All measurements were completed in a double-blind manner.

In accordance with previously-used methodology (Chen et al., 2020), intestinal mucosal damage was assessed using an enhanced version of the Chiu score (Chiu et al., 1970). A five-point scale was used to assess alterations in the villi and glands of the intestinal mucosa in a blinded manner. The grading system for mucosal changes was: grade 0, normal mucosa; grade 1, emergence of the subepithelial Gruenhagen’s space at the apex of the villus; grade 2, expansion of this area accompanied by a modest elevation of epithelial lifting; grade 3, extensive epithelial lifting with some villi becoming denuded; grade 4, denuded villi accompanied by dilated capillaries; and grade 5, degeneration of the lamina propria, occurrence of ulceration, and presence of bleeding.

### 2.4 Analysis of intestinal ultrastructure using TEM

Ileal samples were immersed in pure acetone for 20 min for observation using TEM. The specimens were immersed in a solution of pure acetone and Spurr’s resin in a ratio of 1:1 for 1 h, followed by immersion in a ratio of 1:3 for 3 h. The specimens were then embedded in Spurr’s resin and incubated overnight. The specimens embedded in resin were subjected to heat treatment at 70°C for 9 h to induce solidification of the resin. The specimens were sectioned and stained with uranyl acetate and alkaline lead citrate for 15 min. The cells were then visualized using TEM (Hitachi, Tokyo, Japan). To quantify the autophagosomes and autolysosomes, three samples were included in each group with each sample consisting of 8–10 micrographs. For each mouse, 10 random fields of vision were examined and counted in a blinded manner.

### 2.5 Detection of ileal tight junction proteins

#### 2.5.1 Immunohistochemical (IHC) staining

Paraffin sections were obtained using the same method as that used for H&E staining. After heating at 60°C for 1 h, the slices were dewaxed using xylene. The samples were then rehydrated using a reverse ethanol gradient. Antigen retrieval was performed using microwave boiling (10 mM citrate buffer, pH 6.1). The sections were extracted after natural cooling to room temperature (RT) and then subjected to three washes using 0.01 mol/L phosphate-buffered saline (PBS) at a pH of 7.4, with each wash lasting for 5 min. Horseradish peroxidase (HRP)–DAB staining was performed using an SP Rabbit & Mouse HRP Kit (DAB) according to the manufacturer’s instructions (CWBIO, Beijing, China). Before adding the primary antibodies, a hydrophobic barrier was drawn around the slices using a hydrophobic pen. The sections were incubated in a humidified chamber at 4°C with primary antibodies against zonula occludens-1 (ZO-1; 1:100 Proteintech, Wuhan, China) and occludin (1:200, Abcam, Cambridge, MA, United States), at a dilution of 1:200. Nuclei were counterstained with DAPI following the manufacturer’s instructions. The methodology employed in this study is consistent with the approach described by Zhao et al. (2021). Optical density measurements were performed using Image-Pro Plus 6.0 software.

#### 2.5.2 RNA extraction and reverse-transcription PCR (RT-PCR)

TRIzol RNA purification reagent (Invitrogen, Carlsbad, CA, United States) was used to isolate total RNA. The reverse transcription reaction was performed using a Reverse Transcription Kit (Takara Biotechnology, Kusatsu, Japan), according to the manufacturer’s instructions. Expression primers for ZO-1, occludin, and GADPH were designed using Primer 6.0 software (Premier, Toronto, ON, Canada). The primers used for RT-PCR are listed in Table 1. The PCR cycling conditions were: 15 s denaturing at 95°C, 60 s of annealing, and extension at 60°C for 40 cycles. The homogeneity of the PCR products was confirmed using melting curve analysis. The quantitative fluorescence data were determined using the normalization approach and were calculated using the 2^-∆∆Ct^ formula (Livak and Schmittgen, 2001; Rao et al., 2013).

### 2.5.3 Immunofluorescence (IF) microscopy

Immunofluorescence labeling was used to ascertain the location of tight junction proteins, namely ZO-1 and occludin. Paraffin-embedded tissues were used to create sections with a thickness of 5 μm. Following the dewaxing process, the sections underwent permeabilization using citrate buffer for 15 min in a microwave. Then they were subjected to three washes with phosphate-buffered saline (PBS) and blocked with a solution of 5% bovine serum albumin (BSA) diluted in PBS for 30 min at RT. The sections were subjected to incubation with rabbit anti-occludin (1:100, Proteintech, Wuhan, China) and anti-ZO-1 (1:100, Bioss, Beijing, China) at 4°C overnight. Subsequently, they were treated with FITC-labeled goat anti-rabbit (Bioss, Beijing, China) at a dilution of 1:100 for 1 h at RT. The sections were observed using a Leica DM3000 upright microscope (Leica, Wetzlar, Germany).

### 2.6 Detection of autophagic protein expression and autophagic structures

Ileal protein was extracted using a radio-immunoprecipitation assay (RIPA) lysis kit (Solarbio, Beijing, China). The protein concentration was determined using the bicinchoninic acid (BCA) method. Equivalent amounts of total protein were separated using sodium dodecyl sulfate–polyacrylamide gel electrophoresis (SDS-PAGE) and then transferred to polyvinylidene fluoride (PVDF) membranes. The membranes were sectioned based on the molecular weight of the target proteins. Subsequently, the membrane was subjected to a blocking step using a 5% nonfat milk solution for 2 h at RT. Following this, the membrane was incubated overnight at 4°C with primary antibodies targeting GADPH (1:20000, Proteintech, Wuhan, China), Beclin 1 (1:1000, Bioss, Beijing, China), LC3B (1:2000, Proteintech, Wuhan, China), SQSTM1 (1:1000, Abcam, Cambridge, United States), ATG5 (1:2000, Abcam, Cambridge, United States), t-STAT3 (1:2000, Abcam, Cambridge, United States), and p-STAT3 (1:2000, Abcam, Cambridge, United States). Subsequently, the membranes were incubated with horseradish peroxidase-conjugated goat anti-mouse or goat anti-rabbit IgG for 2 h at RT. The membranes were washed three times with Tris-buffered saline and Tween 20 (TBST). The protein bands were identified by exposing the membranes to X-ray film using an eECL Western Blot Kit (CWBIO, Beijing, China). The quantification of protein band densitometric values was performed using Image-Pro Plus 6.0 software.

The ileal samples for TEM were transferred into pure acetone for 20 min. The specimens were placed in a mixture of pure acetone and Spurr’s resin (1:1 for 1 h and 1:3 for 3 h) and then embedded in Spurr’s resin overnight. The specimens in resin were heated at 70°C for 9 h to solidify the resin and were then cut into sections. The sections were stained with uranyl acetate and alkaline lead citrate for 15 min and visualized using TEM (model H-7650; Hitachi Ltd.).

### 2.7 Data analysis

Data were analyzed using GraphPad Prism software (version 7.0; GraphPad Software Inc., San Diego, California, United States). The data are presented as the mean ± standard error of the mean (SEM) of multiple samples; *p* < 0.05 was considered statistically significant. One-way ANOVA and Tukey’s *post-hoc* test was used for the analysis of differences between multiple groups (*p < 0.05, **p < 0.01, ***p < 0.001).

## 3 Results

### 3.1 Matrine inhibits PCV2 replication in the mouse ileum

**Figure 1:**
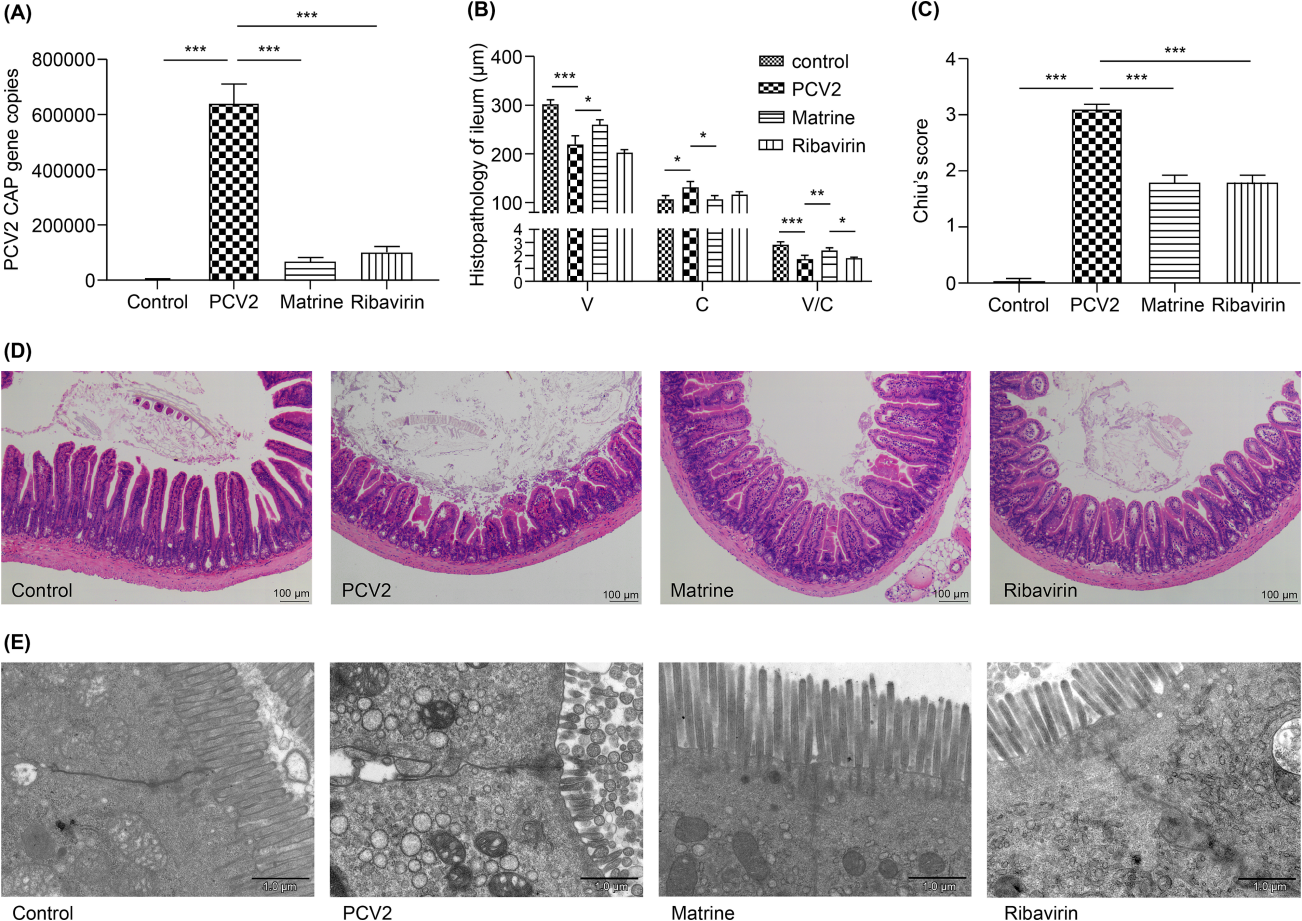
Analysis of the effect of Matrine on the morphology of ileal villi in PCV2-infected mice **(A)** Expression of the PCV2 *Cap* gene in the ileum detected using qPCR. **(B)** H&E-stained ileal villus sections analyzed using ImageJ software. V: villus length, C: crypt depth, V/C: ratio of villus length to crypt depth. **(C)** The assessment of intestinal mucosal damage was performed using the Chiu scoring system (Chiu et al., 1970). **(D)** Histopathological changes in intestinal mucosa (H&E staining). **(E)** Transmission electron micrographs of IEC villi. The results are presented as the mean ± SEM (*n* = 10). ANOVA was conducted, followed by the Tukey *post-hoc* test, to determine the level of statistical significance. *p < 0.05, **p < 0.01, ***p < 0.001. ANOVA, analysis of variance; H&E, hematoxylin and eosin; IEC, intestinal epithelial cells; PCV2, Porcine circovirus type 2; qPCR, quantitative polymerase chain reaction; SEM, standard error of mean.

The qPCR results (Figure 1A) demonstrated an upregulation of ileal *Cap* gene expression in the PCV2 group compared to that in the control group (*p* < 0.001) on day 11 post PCV2 infection (day 6 post drug administration). Furthermore, *Cap* gene expression was decreased in both Matrine and Ribavirin groups compared to that in the PCV2 group (*p* < 0.001). These findings suggest that Matrine possesses the capacity to suppress the replication of PCV2 in the intestinal region of Kunming mice.

Ribavirin is effective against DNA and RNA viruses (Balakrishnan and Mun, 2020; Kim and Lee, 2013), making it an appropriate drug candidate for various viral infections in veterinary medicine (Lee et al., 2020; Wei et al., 2020). In the current study, Ribavirin (40 mg/kg) treatment produced a marked downregulation of PCV2 *Cap* gene expression in the murine ileum. The 40 mg/kg Matrine treatment showed no statistical difference from the Ribavirin treatment.

### 3.2 Matrine attenuates PCV2-induced morphological damage to the ileum

The influence of Matrine on PCV2 infection was investigated in the ileum. There was an increase in villus length and the villi/crypt ratio (*p* < 0.001, *p* < 0.001, respectively; Figure 1B), in parallel to a decrease in crypt depth (*p* = 0.0338, Figure 1B). These findings indicate that the administration of Matrine mitigated the reduction of villus length caused by PCV2 infection (*p* = 0.0283; Figure 1B). Chiu scores were increased in the ileum of PCV2-infected mice (*p* < 0.001; Figure 1C), in accordance with the observed histological changes in the gut and the PCV2 group showed the highest score (*p* < 0.001; Figure 1C).

When comparing the experimental group with the control group, H&E staining revealed compromised integrity of the ileal mucosa in the PCV2 group (Figure 1D), irregular glandular arrangement, shortened and blunted intestinal villi, an enlarged apical gap of the villi, a mucosal layer separated from the lamina propria, a congested lamina propria, and a large accumulation of erythrocytes. This observation indicated that infection with PCV2 resulted in ileal injury in Kunming mice. In the Matrine group, there was partial restoration of the morphology and dimensions of villi, together with a separation observed between the epithelium and lamina propria.

In ileal segments derived from the mice in the control group, the tight junctions located underneath the microvilli appeared narrow and remained intact (Figure 1E). However, expanded pericellular gaps, regions of rarefaction, and vanishing or inflated microvilli were observed in the PCV2 group. The application of Matrine resulted in a more compact arrangement of intercellular membranes. The microvillous structure exhibited a high level of preservation with no marked deformation or effacement. Ribavirin therapy resulted in partial alleviation of the damage observed in the ultrastructure of the ileum in the PCV2 infected mice, characterized by the presence of sparsely and irregularly-distributed microvilli.

### 3.3 Effect of Matrine on tight junction protein expression in PCV2-infected mice

**Figure 2:**
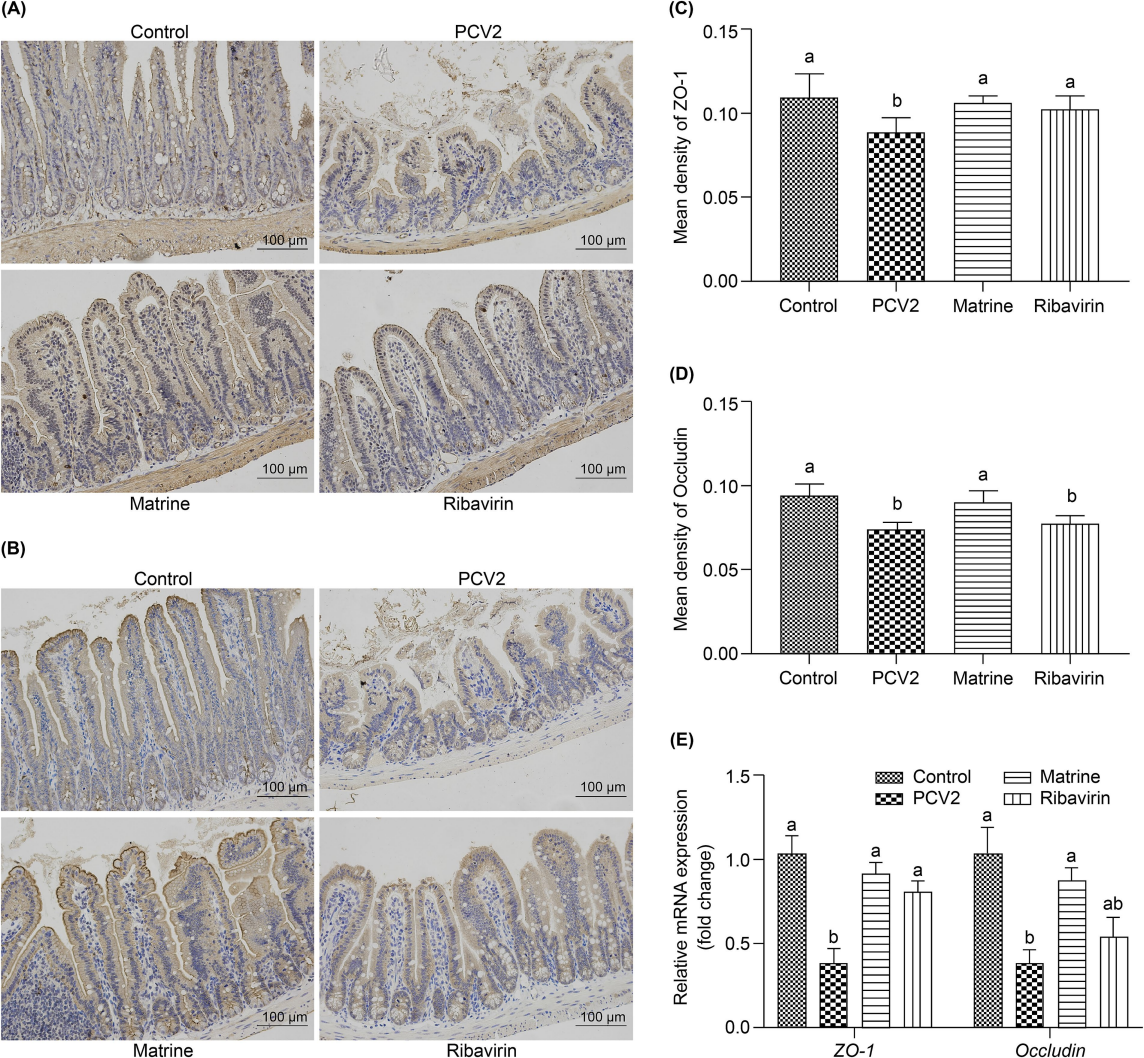
Effect of Matrine on the expression of tight junction-related proteins in the ileum of PCV2-infected mice. **(A, B)** Immunohistochemistry (IHC) of tight junction-related protein in the murine intestine. **(C, D)** Quantitative analysis of IHC-stained images. **(E)** mRNA expression level of tight junction-related proteins in the intestine. The results are presented as the mean ± SEM (*n* = 10).*p < 0.05, **p < 0.01, ***p < 0.001. PCV2, Porcine circovirus type 2; SEM, standard error of mean.

The tight junction protein expression was evaluated using IHC (Figures 2A,B). The PCV2 group had the lowest ZO-1 (compared to the Control and the Matrine groups, *p* < 0.001; compared to the Ribavirin group, *p* = 0.001; Figure 2C) and occludin (compared to the Control and the Matrine groups, *p* < 0.001; compared to the Ribavirin group, *p* = 0.5522; Figure 2D) protein expression. The PCV2 group tended to have the lowest occludin protein expression, whereas Matrine treatment induced upregulation of ZO-1 (*p* < 0.001; Figure 2C) and occludin (*p* < 0.001; Figure 2D) expression.

**Figure 3:**
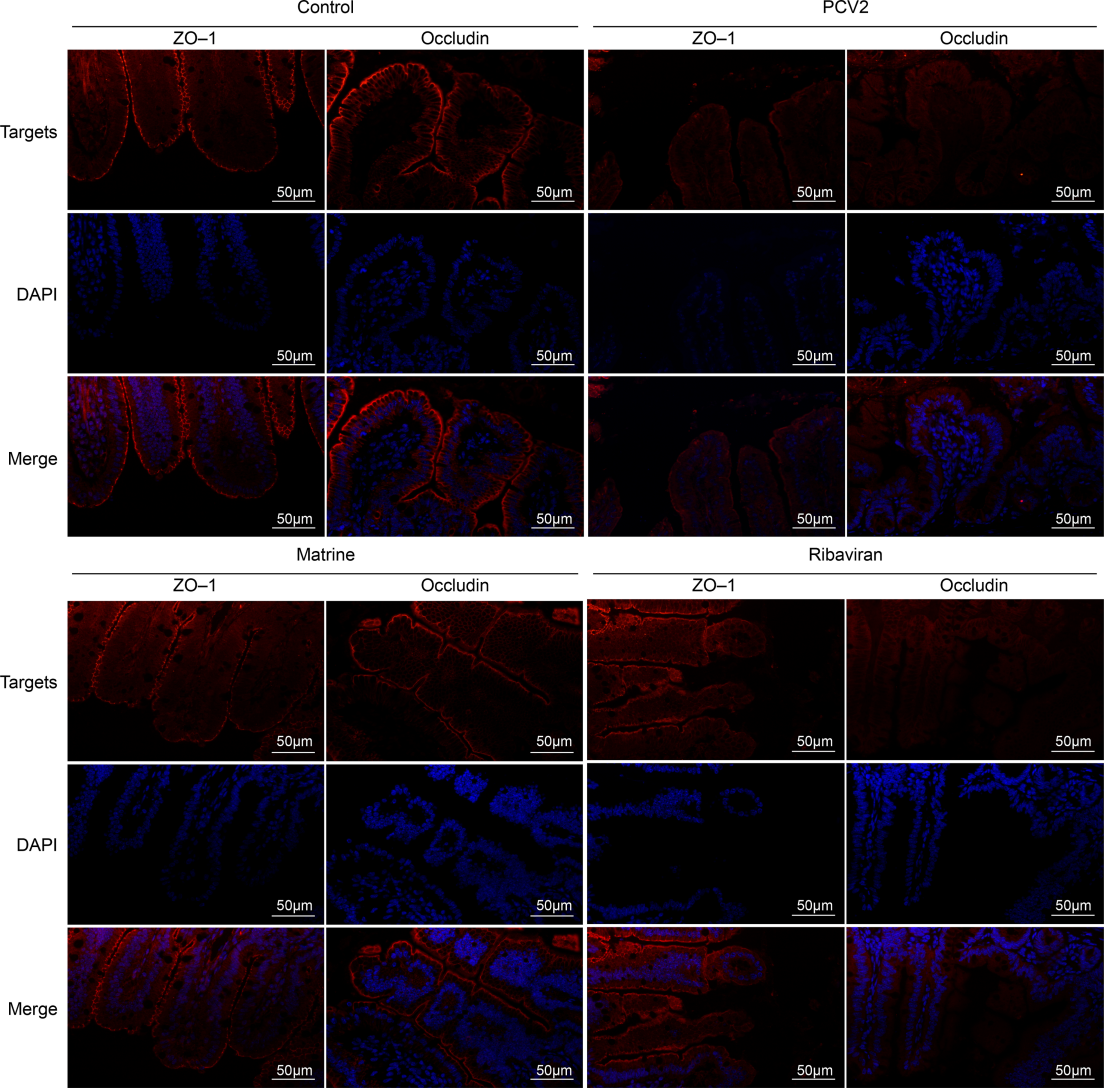
IF staining for ileal ZO-1 and occludin. Nuclei were stained with DAPI, resulting in a blue counterstain. Scale bars = 50 μm. IF, immunofluorescence; ZO-1, zonula occludens-1.

The expression of tight junction-related genes in the ileum was analyzed using qPCR (Figure 2E). PCV2 infection downregulated the mRNA expression of *ZO-1* (*p* < 0.001) and *occludin* (*p* = 0.0065 < 0.01). ZO-1 mRNA expression increased across both treatment groups (*p* = 0.0002, and *p* = 0.0031 for the PCV2 group, compared to the Matrine and the Ribavirin groups, respectively), whereas an increase in the relative expression of *occludin* mRNA was only noted within the Matrine group (*p* = 0.0423). Tight junction protein expression was also assessed using IF staining, and the distribution pattern was consistent with IHC staining results (Figure 3).

### 3.4 Effect of Matrine on modulation of autophagic structures and autophagy-related protein expression in the intestine of PCV2-infected mice

**Figure 4:**
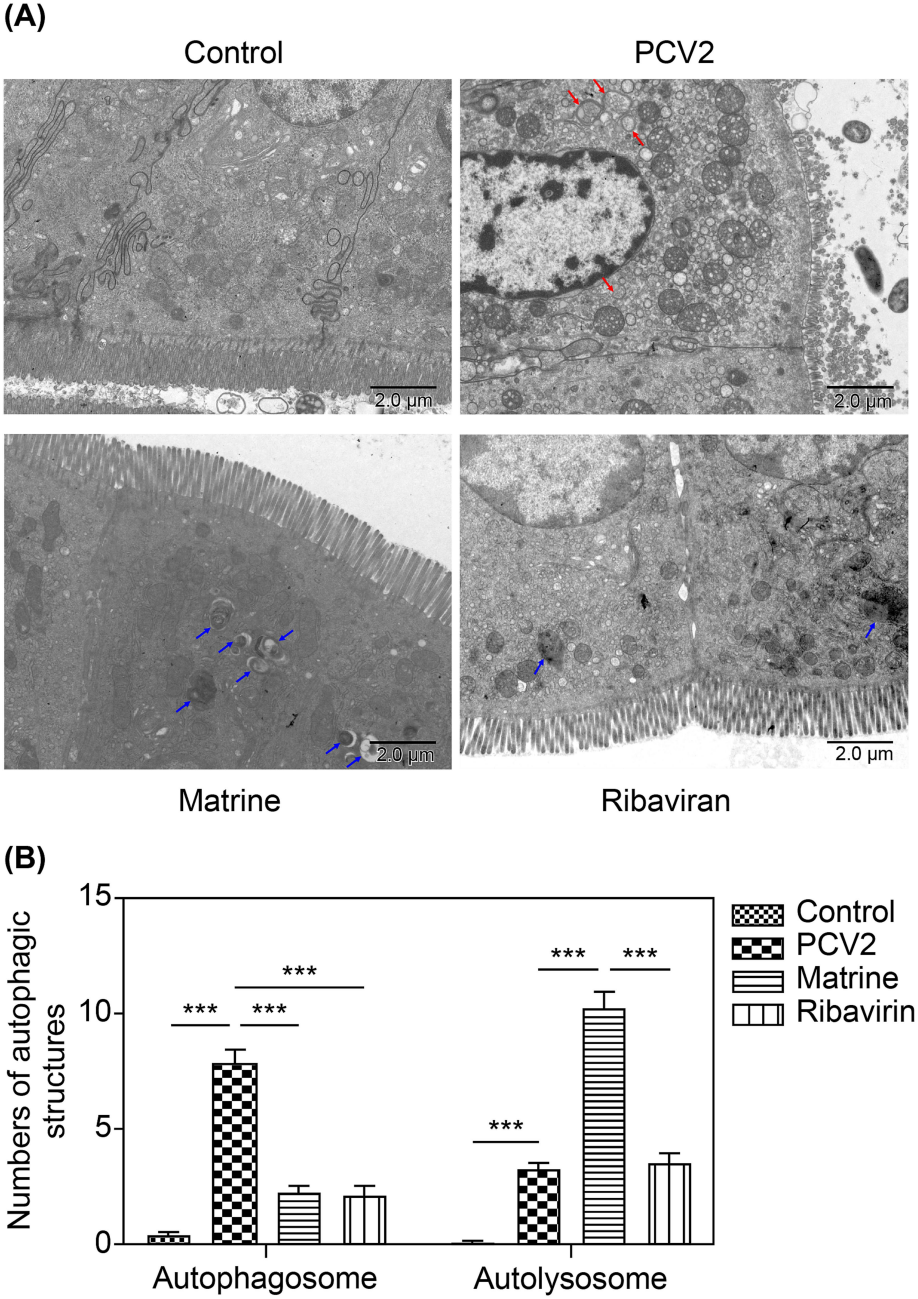
TEM observation of the effect of Matrine on ileal autophagic structures of PCV2-infected mice. **(A)** The representative TEM images of ileal IECs in the control, PCV2 model, Matrine treatment, and Ribavirin treatment groups (4,000× magnification, scale bars = 2 μm). Red arrows indicate autophagosomes, which are characterized by a double membrane and contain multiple small vacuoles. Blue arrows indicate autolysosomes, which are characterized by a single limiting membrane with a higher electron density and amorphous heterogeneous content. **(B)** Quantitative approach for analyzing the abundance of autophagosomes and autolysosomes. Three mice were included in each experimental group, with 10 different fields imaged per tissue sample. IEC, intestinal epithelial cells; PCV2, Porcine circovirus type 2; TEM, transmission electron microscopy.

The number of autophagic vacuoles in ileal IECs was investigated using TEM. A larger number of autophagosomes (red arrows, Figure 4A) with a typical, double-membrane, vesicular structure containing undegraded organelles were present within ileal tissue of the PCV2 group than in the control group (*p* < 0.05, Figure 4B). In the Matrine group, there was a reduction in the number of autophagosomes compared to the PCV2 group (*p* < 0.05, Figure 4B). In contrast, the number of autolysosomes (blue arrows, Figure 4A) was higher in the Matrine than in the PCV2 group.

Compared with the control group, the mice in the PCV2 group showed ultrastructural damage in the ileum, characterized by an increase in intercellular space, swelling of mitochondria, and a loss of clarity in cristae and the nuclear heterochromatin border set, endoplasmic reticulum expansion, and vacuolation. Ultrastructural analysis revealed that Matrine mitigated necrotic ultrastructural alterations in the ileum. The Matrine group had intact mitochondrial cristae and only slight mitochondrial damage (Figure 4A). In the Ribavirin group, mitochondria were moderately swollen, with greater mitochondrial intramembrane matrix fading and fewer inner cristae compared to the control group.

**Figure 5:**
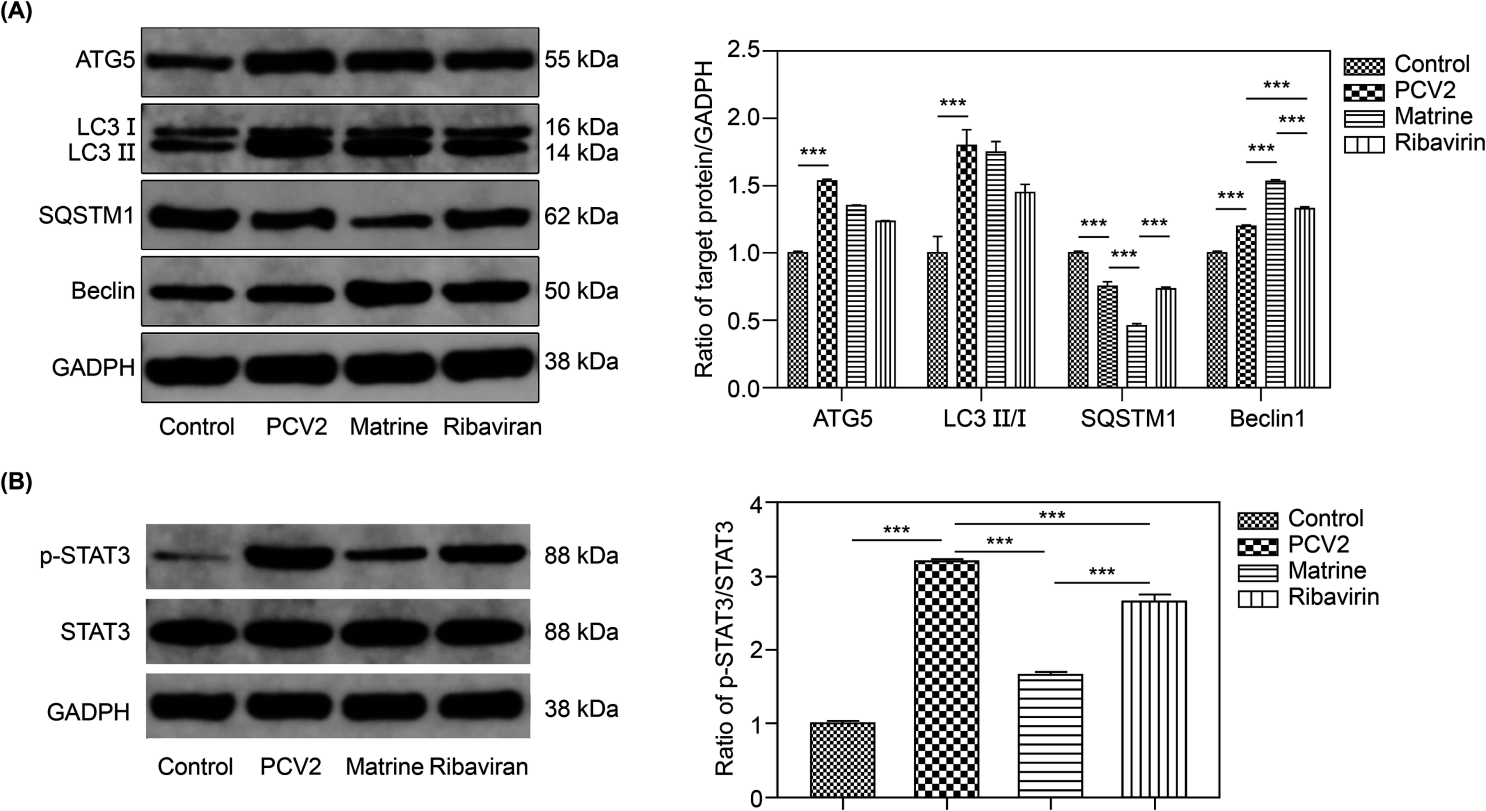
Effect of Matrine on autophagy-related proteins in the ileal tissues of PCV2-infected mice **(A)** Representative western blot bands of autophagy-related proteins and quantitative band intensity analysis. **(B)** p-STAT3/STAT3 ratio. Data are expressed as the mean ± SEM (*n* = 10). *p < 0.05, **p < 0.01, ***p < 0.001. PCV2, Porcine circovirus type 2; SEM, standard error of mean.

TEM can be used to indicate the presence of autophagic structures. To ascertain the potential involvement of autophagy in the protective effect of Matrine on intestinal barrier integrity, the expression of the autophagy markers, LC3II, Beclin-1, and SQSTM1, was examined. To determine the effect of Matrine on autophagy following PCV2 infection, ileal tissue samples were used to assess the levels of the aforementioned proteins across the groups. The ileal ATG5 protein level (*p* = 0.0005, Figure 5A) and the LC3 II/LC3 I ratio (*p* = 0.001, Figure 5A) were higher in the PCV2 than in the control group, indicating that PCV2 infection induced autophagy in the ileum of mice. Furthermore, upregulation of Beclin-1 (*p* < 0.001, Figure 5A) and downregulation of SQSTM1 (*p* < 0.001, Figure 5A) were observed after PCV2 infection (Figure 5A). Compared to the PCV2 group, SQSTM1 expression was reduced (*p* < 0.001, Figure 5A) and Beclin1 expression was increased (*p* < 0.001, Figure 5A) in the Matrine group, and the change in the expression of both proteins was more pronounced than in the Ribavirin group (*p* < 0.001, Figure 5A).

We determined the effect of Matrine on STAT3 protein phosphorylation (Figure 5B). Infection with PCV2 resulted in enhanced STAT3 phosphorylation (*p* < 0.001, Figure 5B). Matrine inhibited STAT3 phosphorylation and activation caused by PCV2 infection (*p* < 0.001, Figure 5B).

## 4 Discussion

In this study, we established a mouse model of PCV2 infection. PCV2 replicated and proliferated in the mouse ileum. The down-regulation of tight junction protein expression caused pathological damage to the intestinal mucosa and a decrease in the structural integrity of the intestinal barrier. The host usually initiates an autophagy program to resist virus invasion. In contrast, PCV2 viruses avoid clearance by facilitating the assembly of LC3+ autophagosomes, increasing autophagosome accumulation, and inhibiting the expression of autophagy maturation-related proteins. We administered Matrine to PCV2-infected mice. The viral load assay showed a substantial decrease in *Cap* gene expression. Morphology results showed that Matrine attenuated PCV2-induced intestinal damage. Matrine reversed PCV2-induced autophagosome accumulation to resist viral hijacking of the autophagy process in intestinal cells by inhibiting STAT3 phosphorylation, upregulating Beclin1 protein expression, promoting viral proteins to be degraded by autolysosomes, and reversing PCV2-induced autophagosome accumulation to attenuate intestinal damage.

Gut morphology reflects the ability of the intestine to absorb nutrients. Intestinal morphological alterations caused by PCV2 included elevation of the epithelium at the apex of the villus, villus atrophy, and hemorrhage in the lamina propria. Such mucosal modifications may negatively affect intestinal nutrient absorption function. Matrine enhanced morphometric outcomes in the ileum, specifically in terms of elevating villus height, reducing crypt depth, and optimizing the villus-to-crypt ratio. IECs generated from stem cells situated in the lower region of the crypt undergo periodic turnover, whereby they depart from the villus tip; subsequently, these IECs are replenished by newly-generated cells that migrate upwards from the crypts (Mowat and Agace, 2014). The deepening of the ileal crypts in the intestine of PCV2-infected mice therefore indicates increased cell proliferation, a reduction in the maturation rate of intestinal epithelial cells, and a decrease in intestinal secretion. Matrine enhanced the differentiation potency of crypt stem cells, as evidenced by the substantially increased villi/crypt ratios in this treatment group. Overall, Matrine not only improved intestinal morphology but also enhanced the renewal capacity of intestinal cells, thus counteracting the impairment of intestinal function by PCV2 infection.

The intestinal barrier, which serves as the primary location for the interaction between pathogenic microbes and toxins, such as PCV2, consists of linked epithelial cells that form an intercellular junctional complex. The IECs, together with tight junctions between the cells, form the intestinal mechanical barrier and regulate its paracellular permeability (Chelakkot et al., 2018). This complex encompasses many types of intercellular junctions, including gap, adherens, and tight junctions, as well as desmosomes. Tight junctions are fundamental regulators of paracellular route permeability (Buckley and Turner, 2018). ZO-1 and occludin are integral tight junction proteins and are crucial for maintenance of the intestinal epithelial cell barrier (Niessen, 2007). Occludin interacts with the intracellular protein, ZO-1, thereby facilitating binding to its backbone protein (Al-Sadi et al., 2011). Matrine attenuated the PCV2-induced downregulation of ZO-1 and occludin in the current study. Consistent with our findings, it has been shown that *Astragalus* polysaccharide (AP) and Matrine ameliorate histopathological changes in rats with ulcerative colitis, with increased expression of ZO-1, occludin, and TFF3 in lung and colon tissues (Yan et al., 2020). The current study revealed the remission of ultrastructural morphological indicators in PCV2-infected IECs within the ileum after Matrine treatment, while attenuating PCV2-induced down-regulation of ZO-1 and occludin and restoring intestinal barrier function.

PCV2 infection contributes to a substantial disturbance in mucosal mechanical barrier function. Autophagy can support intestinal luminal IEC–dendritic cell interactions and prevent alterations in barrier permeability by regulating tight junctions, and a deficiency in autophagy during intestinal disease may lead to elevated paracellular permeability (Asano et al., 2017; Foerster et al., 2022). PCV2 and PRRSV were shown to utilize autophagy to enhance their proliferation (Lv et al., 2020; Wang et al., 2019; Zhai et al., 2019; Zhu et al., 2012b). Lv et al. (2020) reported that the PCV2 ORF5 protein induced autophagy in PK-15 cells and facilitated PCV2 replication primarily via activation of the PERK–eIF2α–ATF4 and mTOR–ERK1/2–AMPK signaling pathways. In the IECs of PCV2-infected mice, we observed a considerable number of double-membrane structures, a typical structure thought to be the autophagosome of early autophagic vesicles that encompasses undegraded organelles, such as mitochondria, endoplasmic reticulum fragments, and cytoplasmic components (Klionsky et al., 2016; Papinski et al., 2014). The accumulation of autophagosomes is the result of either increased autophagosome formation or impaired degradation (Benyounès et al., 2011). The process of autophagosome elongation and maturation relies on the ubiquitin-like protein molecule, LC3, which is involved in the formation of the double-membrane structure of autophagosomes (Fujita et al., 2008; Klionsky et al., 2007; Mizushima, 2020). After PCV2 infection, the up-regulation of ATG5 expression promoted the conversion of LC3 I–LC3 II, which may be related to impeded autophagosome destruction.

A large number of autolysosomes, as opposed to autophagosomes, appeared after 6 days of Matrine treatment, and were no longer intact due to degradation of their cytoplasmic material. As an adapter protein, SQSTM1 transports damaged or aged organelles and proteins to the autophagosomes for degradation (Pankiv et al., 2007). The present study demonstrated that SQSTM1 was degraded concurrently with the progression of the autophagy pathway following the administration of Matrine to PCV2-infected mice, confirming that Matrine reduced the accumulation of autophagosomes caused by PCV2 infection. Viral infection can inhibit the phagocytosis of infected cells by interfering with the degradation of autophagic cargo in the normal autophagy–lysosome pathway through inhibition of autophagic vesicle fusion with late endosomes and lysosomes (Choi et al., 2018).

Miller et al. (2020) reported that the mechanism by which viruses inhibit the progression of the autophagy–lysosome pathway is through direct or indirect inhibition of the expression of Beclin1, a host protein that promotes the fusion of autophagosomes with lysosomes. Matrine-induced Beclin1 upregulation leads to its separation from Bcl-2/Bcl-xL and subsequent autophagy induction, which greatly inhibits the development of hepatocellular carcinoma xenografts (Yang and Yao, 2015). In the current study, Matrine treatment increased the expression level of Beclin1 protein and promoted the autophagy process. The negative correlation between Beclin1 and SQSTM1 also indicated enhanced autophagy following Matrine treatment.

As a signal transducer and activator of transcription, STAT3 plays a negative regulatory role in autophagosome degradation (You et al., 2015). Beclin1 is a direct transcriptional target of STAT3, and stimulation of the JAK2/STAT3 signaling pathway directly impedes the transcriptional activity of the autophagy regulator, Beclin1. This results in the inhibition of autophagy and induction of cell death in intestinal cells (Liu et al., 2022b). We demonstrated previously that Matrine reduces the production of the inflammatory cytokine, interleukin-6 (IL-6), in mice infected with PCV2 (Sun et al., 2022). STAT3 is a key downstream transducer of IL-6 receptor activation (Wang et al., 2013), which might be related to the modulation of STAT3 activation exerted by Matrine. The results of the current study demonstrate that PCV2 infection may inhibit Beclin1 protein expression by promoting phosphorylation of STAT3 protein, which Matrine intervention inhibits. This promotes Beclin1 protein expression to induce fusion of autophagosomes and lysosomes. Autophagic vesicle degradation is ameliorated to promote viral clearance by the organism. Matrine had a greater modulatory effect than Ribavirin.

In the present study, we identified an important role for Matrine in host intestinal defense against PCV2 infection. We speculate that autophagy is an important countermeasure for Matrine to enhance the intestinal barrier defense response, leading to the finding that Matrine regulates autophagy in ileal epithelial cells in a PCV2-infected murine model. Further research is required to elucidate the dynamic monitoring of the autophagy process *in vivo*, as well as the use of autophagy activators and inhibitors to investigate the protective effect of Matrine in activating autophagy in host cells hijacked by PCV2 and its immunomodulatory effects. Our findings contribute to the understanding of the novel mechanism of the antiviral activity of Matrine and provide a strategy for host-directed, innovative drug discovery by targeting autophagy activation in intestinal IECs and blocking viral hijacking of autophagy in host cells.

## Data Availability

The raw data supporting the conclusions of this article will be made available by the authors, without undue reservation.
